# Accelerated Digitalization of the Epidemiological Measures: Overcoming the Technological and Process Complexities of Establishing the EU Digital COVID Certificate in Slovenia

**DOI:** 10.3390/ijerph192114322

**Published:** 2022-11-02

**Authors:** Dalibor Stanimirovic, Lucija Tepej Jocic

**Affiliations:** 1 Faculty of Public Administration, University of Ljubljana, 1000 Ljubljana, Slovenia; 2 National Institute of Public Health, 1000 Ljubljana, Slovenia

**Keywords:** COVID-19 pandemic, EU Digital COVID Certificate, eHealth infrastructure, technology and processes, complexities, Slovenia

## Abstract

Objective: In order to facilitate free movement of EU citizens during the COVID-19 pandemic, in early 2021 the European Commission proposed the establishment of an EU Digital COVID Certificate. By 1 July 2021, all EU Member States have successfully implemented the EU Digital COVID Certificate. The technological and procedural complexities encountered while establishing the EU Digital COVID Certificate in Slovenia are reviewed in this article. Methods: This research employs a case study methodology. Controlled focus group sessions comprising eighteen eminent experts (not including medical and other expert groups involved in the epidemiological measures) in charge of the EU Digital COVID Certificate and other national eHealth services in Slovenia were used as the primary data collection technique. Focus group discussions were preceded by an all-encompassing review of the literature and the examination of numerous materials covering the EU Digital COVID Certificate-related content. Results: The study findings reveal that the technological and process complexities are associated with the fragmented data sources and complicated and abundant business rules used for the generation and verification of the EU Digital COVID Certificate. However, despite the technological, process and other impediments that arose during the establishment of the EU Digital COVID Certificate in Slovenia, it can be argued that the approach used and stakeholder commitment, especially in critical pandemic conditions, offered the much-desired harmonisation and application of this digital service at the EU level. Conclusions: The study highlights the importance of a sound and coherent model for the impending establishment of cross-border eHealth services and suggests that the ad hoc implementation of such challenging and delicate digital solutions in the future will only be feasible with the prior construction of robust and interoperable digital health infrastructures across the EU Member States.

## 1. Introduction

The EU Digital COVID Certificate (DCC) [1] was implemented in all EU Member States on 1 July 2021. It is set up on the uniform structure and central digital platform established by the European Commission, and supports the issuance and verification of interoperable certificates of the COVID-19 tests, vaccinations, and recovery [2]. The DCC is equally recognized and approved in all EU Member States, enabling the unhindered border crossing and harmonized elimination of potential travel restrictions and ensuring synchronized approach to issuing and verification. All DCC holders (citizens of the EU Member States and nationals from third countries legally residing in the EU) should be exempt from restrictions on free movement under the pandemic health conditions, and visitors are to be treated equally as the residents of the visited country. The DCC issuing criteria is defined by the regulation of the European Parliament and the Council of the EU [3], while the technical specifications for the establishment of the DCC system are proposed by the European Commission [4]. The whole DCC concept including issuing and verification conditions is characterized by complex business rules depending on the timeline and sequence of vaccination and test events. Business rules represent a set of predefined scenarios and related controls that allow the issuance and verification of the DCCs in accordance with the epidemiological directives. Compliance with business rules in the issuance phase results in the issuance or non-issuance of the DCC, while the compliance with business rules in the verification phase results in the acceptance or invalidation of the DCC. However, regardless of the uniform regulatory and technical framework, EU Member States are still eligible to enforce legal actions autonomously to a certain degree, so national authorities may still apply different travelling and border entry conditions [5,6]. Obligations of healthcare providers with regard to electronic records of vaccinations and test results are subject to national regulations [7,8] and therefore may differ between the Member States. The DCC holds vital information: name, surname, date of birth, date of issue, information on the negative test results, vaccination against the COVID-19, recovery from the COVID-19, and a distinctive certificate identifier. The set of data and their content in the DCC is mandated, while the form of the DCC itself was only drafted by the European Commission, and the Member States retained the right to their own design images. The DCC is issued only for COVID-19 and contains a restricted set of information that cannot be preserved by the visited countries. During the verification stage, only the authenticity and validity of the DCC are inspected, and the signatory authority that issued the DCC is checked. The issuing Member State is solely responsible for the data retention, accuracy, and integrity of the DCC information.

The DCC project initiated by the European Commission and the EU Member States represents one of the unplanned measures to restrain the COVID-19 pandemic in the last year and a half. With the announcement of the DCC and its introduction in the EU as well as in some other countries around the world, many questions and dilemmas arose regarding its legitimacy, effectiveness and implications for citizens, public health, and broader socioeconomic aspects. As a result, a certain amount of published research has appeared, which is understandably limited both in terms of number and content due to the recent appearance of the DCC. Although the contents of the research often intertwine and touch on diverse but related issues, in our opinion they can generally be classified into three larger groups with common characteristics, namely epidemiological concerns, attitudes to vaccination, and health outcomes [9,10,11]; public health, healthcare and social policies and measures [12,13,14], and legal aspects, democratic values, and human rights [15,16,17]. Even if some articles touch on individual digital tools for managing the COVID-19 pandemic, the content of the research mostly refers to applications for contact tracing, international comparisons of the DCC projects and implementation approaches, and analysis of security or privacy of the end-user’s personal data [18,19,20,21]. In this sense, this article reveals the aspects and challenges of establishing the DCC which have not yet been sufficiently explored. Therefore, this contribution represents one of the few attempts in the field and can serve as a starting point for all future studies of the technological, process and other potential complexities (infrastructural, architectural, organizational) which may arise during the establishment of the DCC or similar initiatives.

As noticed in some articles, the terms and concepts concerning the COVID-19 certificates have a rather arbitrary and interchangeable meaning, which significantly hinders their characterization and distinction from other vaccination-related documents. The latter reasons considerably hamper the research in the field and prevent the consistent methodological approach in assessment of the technological, process and other aspects concerning the deployment of the DCC in national digital infrastructures. However, regardless of the research perspective, virtually all articles underscore that the main goals of the DCC and other related concepts and initiatives in healthcare crises should be focused on the health benefits of patients and effectiveness of the healthcare systems.

From the very conception of the DCC initiative, it was clear that it was a very large and demanding project. The latter applies both to the amount and diversity of data needed for issuing and verifying the DCCs, as well as to the technological and process requirements that need to be harmonized and implemented at both national and EU level [22]. In addition, it is important to emphasize the enormous risk posed by such a controversial initiative in many ways, which actually restricts citizens’ fundamental rights in terms of free movement between EU countries and whose failure could have profound and wide-ranging consequences for the EU citizens and different social subsystems in the Member States [23]. On the other hand, there were also serious concerns about how this kind of pioneering cross-border project on the whole EU territory would turn out, especially in such an important area as the health of citizens and in such disturbing circumstances like the COVID-19 pandemic [24]. Due to all of the above and the alarming epidemiological trends in EU countries, the expectations of the public were enormous, not to mention the very short time frame, which placed additional pressure on the National Institute of Public Health (Nacionalni Inštitut za Javno Zdravje, hereinafter: NIJZ) in Slovenia that was in charge of implementing the entire DCC system. The NIJZ runs the national eHealth solutions and is the only authorized DCC issuer in Slovenia. The digital solution enabling the generation of the DCC is based on the records kept in the Central Registry of Patient Data (CRPD), the core platform for exchange of Electronic Health Records (EHR). The DCC contains a digital seal, which confirms its validity and authenticity, and a QR code that holds the essential information and facilitates automatic verification. The DCC is provided for all residents of Slovenia, and online access is enabled for patients and healthcare professionals. Offline paper copies are available, as well as offline storage in mobile wallet application.

As we can see, the project of establishing the DCC system had to overcome numerous and complex obstacles and build a solution that efficiently draws data from various sources of the national digital health architecture and successfully communicates with central infrastructure building blocks at the EU level. In view of that, this article centres on a review of the technological and process complexities identified in establishing the DCC in Slovenia.

## 2. Methods

### 2.1. Research Design

This article applies a case study methodology including structured focus group discussions as the main data collection technique to investigate the technological and process complexities identified in establishing the DCC system in Slovenia. Experts from the NIJZ, government officials from the Ministry of Health, and specialists from external IT vendors took part in twenty focus group sessions carried out from October 2021 to March 2022 (each videoconference session lasting about two hours). Focus group discussions with prominent experts in the field of digital health and public health were preceded by a wide-ranging review of the literature and the exploration of different sources containing the DCC-related materials. A conventional content analysis was employed later in the process to scan and interpret the catalogued transcripts of the focus group discussions. Since this study is mostly exploratory in nature, especially the part concerning the unique approach of the DCC, quantitative empirical approach could not provide credible results. The choice of the research methodology was based on the particularities and specifics of the research subject, availability of the evidence, and the novelty of the DCC concept [25,26]; the research approach used was deemed to be the most favourable one.

### 2.2. Sample

The choice of the prospective focus group participants was based mainly on their experience and expertise. Proficient understanding of the architectural, infrastructural, data, technological, process, and ecosystemic characteristics of the digital health landscape in the country by the carefully chosen focus group participants was anticipated to ensure the validity and reliability of their opinions and recommendations, as well as to enable their productive participation throughout the study [27]. Accordingly, a non-random sampling approach was applied to ensure a representative sample of experts that meet the necessary conditions [28,29]. The response rate reached 100% since all summoned experts confirmed their participation and took part in the focus group discussions. Quota of experts was determined after reaching saturation point [30]. Ultimately, eighteen renowned experts from the field joined the research and participated in the focus group sessions (not including medical and other expert groups involved in the epidemiological measures). The participants were characteristically eHealth managers, ministry officials and engineers occupying top positions, most of them from the NIJZ (10), Ministry of Health (3), and external IT vendors (5). They were typically aged between 40 and 60 years, and the men to women ratio was 12:6 (67% men and 33% women). Their competences and active collaboration during the entire study was supposed to guarantee the overall integrity of the study and, above all, impartiality of the research findings.

### 2.3. Data Collection

The focus group sessions were conducted by the authors via teleconference platform in compliance with social distancing recommendations at the time. The motive and goals of the study were explicated to all participating experts in order to illuminate the fine points and possible ambiguities related to their tasks. All participating experts provided informed consent and were assured confidentiality and anonymity. The invited experts actively participated throughout the entire study, so additional authorization of their interpretations and views was not needed. Focus group participants had two tasks within the scope of the study. First, participating experts had to provide a wide-ranging analysis of the entire digital health infrastructure and eHealth landscape in Slovenia. Second, based on their knowledge and experience in the field, they had to outline the technological and process complexities concerning the establishment of the DCC in Slovenia.

Following an extensive review of the literature, EU guidelines and technical specifications, a thorough analysis concerning the establishment of the DCC in Slovenia was conducted with the participating experts. The perceptions and estimations of the focus group participants were substantiated by the business intelligence and statistical modules and data from the DCC application and other eHealth solutions [31]. The responses and argumentations from the focus group discussions were later converted into transcripts and data tables, which were eventually archived and registered. The methodological approach used and data collected ultimately facilitated the qualitative and quantitative analyses presented in this study.

The differences between quantitative and qualitative research methods are multifaceted. They originate from the theoretical, philosophical and value aspects of the study, and go all the way to more tangible areas that touch on populations and sampling, techniques of data collection and analysis, types of research design, and methods of inference [32]. Quantitative research is based on a representative sample with the aim of generalizing the research findings to the population, while qualitative research deals with the study of individual cases or phenomena. Accordingly, typical data collection techniques are used. In quantitative research, these are structured data, rating scales, and closed-type questions, which, through quantification, enable the performance of statistical analyses. Qualitative research, on the other hand, is characterized by unstructured techniques of data collection such as review and analysis of literature and documents, focus groups, observations, semi/structured interviews, etc. [33]. As this study deals with the new DCC concept, which represents a unique case in terms of its purpose and form, and all this in a field where there is almost no structured information and quantified data, the qualitative research approach was chosen to be the primary one.

### 2.4. Data Analysis

The analysis of the data obtained was performed using a conventional content analysis in accordance with the content analysis methodological guidelines [34]. Implementation of the content analysis was based on codification of the key constructs within specific dimensions highlighted in the documented transcripts. The coding classifications were acquired from the earlier literature review and straight from the focus group discussions [35]. The essential purpose behind the codification process is to assess the claims, assertions and arguments of the focus group participants by finding confirmatory references and/or quotations in the documented discussions (transcripts). A concluding content analysis of the transcripts was conducted independently by coders (authors) in order to strengthen the impartiality and dependability of the results, while all identified discrepancies were re-evaluated by the coders cooperatively [36].

The successful completion of the data analysis was greatly supported by the use of the suitable methodological approach including the appropriate data collection procedure. The applied research methodology, which includes a combination of different data collection approaches and techniques, was of crucial importance for obtaining relevant data and their subsequent analysis. The latter offered a useful framework for the synthesis and interpretation of the collected data, which finally made it easier to present the findings based on solid evidence and adopt credible conclusions.

## 3. Results

### 3.1. Technological Complexities—The Slovenian eHealth Architecture and the CRPD

**Figure 1:**
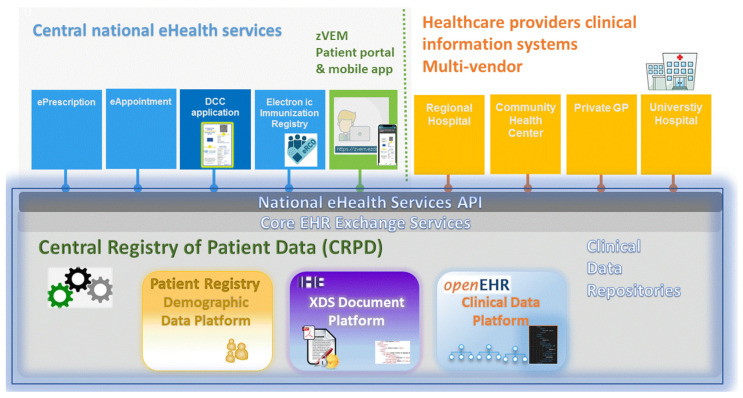
The Slovenian eHealth architecture and the CRPD.

The CRPD is the fundamental platform of the Slovenian eHealth architecture. It is based on the IHE XDS [37] and OpenEHR [38] standards and facilitates sharing of the EHR on a national level (Figure 1). Healthcare providers are connected via standardized Application Programming Interface (API). The CRPD consists of three building blocks:

Integrated Healthcare Enterprise (IHE) document platform providing basic functions for storage and exchange of a variety of healthcare documents and records. It is accessible to information systems of all public and private healthcare providers via a proprietary API called Integrated Healthcare Adapter (IH Adapter), which is customized according to regulative and organizational requirements of the Slovenian healthcare. Healthcare providers are free to choose any clinical information system as long as it is adequately configured and interoperable with the API.

Open EHR data platform for storage of structured clinical data. Upon submission of a structured clinical document, the pertaining data record is stored in the OpenEHR database, contributing to the health data repository.

Patient Demographics Platform (Patient Registry), which is connected to public registries. The Patient Demographics Platform contains up-to-date demographic and status information on all citizens and residents of Slovenia. This includes the permanent and temporary address, information on next-of-kin and health insurance.

The three building blocks are orchestrated by the CRPD application, providing a comprehensive set of EHR management services. The architecture of the Slovenian eHealth enables it to harness the full potential of test and vaccination records. Upon entering a single vaccination record in the physician’s clinical information system, the vaccination data is submitted to the CRPD and is instantly available to all healthcare professionals. Moreover, it is automatically transferred to the Electronic Immunization Registry. In case of the COVID-19 vaccination, the record is immediately available for issuing the DCC of vaccination. A similar algorithm applies to COVID-19 test results. Upon entering a test result in the local information system at the healthcare provider, the test record is instantly available for issuing the DCC of negative test or recovery in case of positive test. At the same time, positive test results are automatically captured in the national COVID-19 registry.

### 3.2. The DCC Application

**Figure 2:**
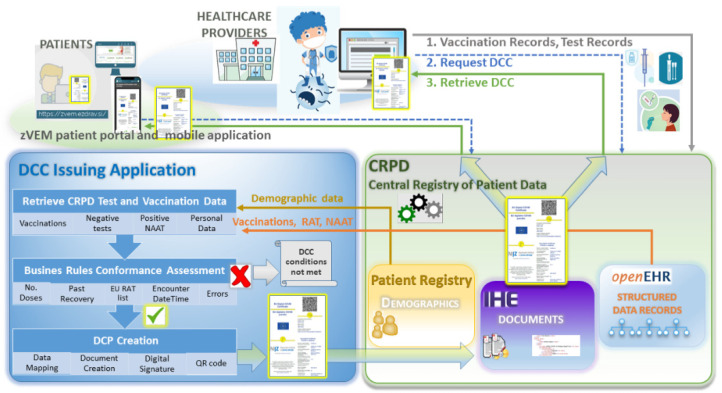
The DCC system architecture.

The DCC application was introduced as a new eHealth service aimed for issuance of the DCC based on the electronic record of test results and vaccinations stored in the CRPD. It is located on top of the core CRPD components and consists of the functional elements and building blocks illustrated in Figure 2. The DCC application performs the retrieval of data on test results and vaccinations from the CRPD, validation with regard to the applicable business rules, and the generation of the DCC document including a digital signature and QR code. Records of the COVID-19 vaccinations, rapid antigen test (RAT) results and nucleic acid amplification test (NAAT) results present data sources for the generation of the DCC. The DCC is obtainable instantly upon the transfer of the relevant test result or vaccination record. Query mechanisms for vaccinations and the COVID-19 test results are separated from the generic CRPD services, extracting relevant records in the given timeframe and ensuring high performance with minimal extra load to the core components.

A demand for the DCC is initiated when a common query for documents in CRPD is performed, and the DCC is simply added to the list of patient’s healthcare documents. When a user activates an action for retrieval of the DCC, the DCC application automatically retrieves the data from the CRPD, extracts the data with regard to the business rules, and generates the DCC according to the data presently available in the relevant timeframe. Compliant with the technical requirements [4], the DCC is digitally signed by the issuer’s digital certificate and equipped with a QR code, enabling authorized personnel to perform automatic verification. If issuing conditions are not met, an error message indicating the unmet conditions is displayed on the DCC.

The DCC is generated in a form of an On-Demand document, applying one of the basic functions of the CPRD Document Platform (IHE XDS.b integration profile, option On-Demand document [39]). For healthcare professionals, the process of obtaining the DCC is analogous as for any other healthcare document. All Slovenian healthcare providers have instant access to the DCC for any patient through existing local clinical information systems and no modification of their local information systems at points of care is needed. Patients can acquire their own DCC through the zVEM Mobile Application or the zVEM Patient Portal (https://zvem.ezdrav.si, accessed on 25 September 2022). On the other hand, healthcare providers can also obtain the DCCs through the zVEMplus web portal, which is intended for healthcare providers whose local information systems are not yet linked to the CRPD (e.g., pharmacies).

A precondition for the generation of the DCC is duly recorded data, and all healthcare providers are legally obliged to timely submit accurate records of test results and vaccinations to the CRPD. After extracting relevant records, the DCC is generated and digitally signed with the system certificate issued by the NIJZ. An essential component of the DCC is the QR code, which enables authorized personnel to conduct automatic verification using a mobile verifier application.

### 3.3. Verifier Application

The mobile verifier application captures the relevant data from the QR code, and verifies the validity and authenticity of the DCC. If business rules for validity are followed, the DCC is approved; if not, an indicator of non-validity is displayed. The Slovenian mobile verifier application is conformant to the common EU technical specifications [4] and customized with regard to the national regulations [40,41].

### 3.4. Patient Access to the DCC

The zVEM Patient Portal was rolled out in 2017 as a common national patient-facing application for all eHealth services. In 2021, a zVEM Mobile Application was introduced. Besides the DCC, the portal and mobile application enable access to a variety of medical documentation such as Patient Summary, ambulatory reports, consultation notes, digital imaging reports, microbiology laboratory reports, discharge letters, vaccinations, e-referrals, e-prescriptions, etc. [42]. To access zVEM Patient Portal, a digital identity issued by the Trust Service Authority SI-PASS is needed [43]. A real-time-generated DCC can be obtained on request. Additionally, the mobile application provides a secure wallet for offline storage of the DCCs. The storage is performed by simply scanning a valid QR code, enabling storage of the DCCs belonging to family members.

#### Process Complexities—Operational and Organizational Aspects of the DCC Introduction in Slovenia

The first case of the COVID-19 in Slovenia was confirmed at the beginning of March 2020. The pandemic was declared on 12 March 2020, resulting in immediate decline of the functioning of the healthcare system either due to fear of infections or due to epidemiological protocols concerning clinical practices. Physical access to healthcare services was severely limited, and national eHealth services became indispensable for secure access to essential data and for remote communication between healthcare providers as well as between patients and healthcare professionals [44]. The DCC became an essential document providing not only access to healthcare but also workplaces, schools and public venues. Consequently, the zVEM Patient Portal in Slovenia faced an amazing upsurge in the number of registered users and single visits (Table 1).

The interest for DCC and registration to the zVEM Patient Portal reflected in a massive demand for digital identity that could not be processed within regular capacities of state trust service authority SI-TRUST [45]. Mandatory digital ID as a prerequisite to accessing the DCC was initially objected as allegedly too strict and burdensome for citizens, but the NIJZ insisted on retaining the highest authentication standards and seized the opportunity to foster adoption of electronic identification service amongst the widest population. Ministry of Public Administration ensured additional human resources and set up provisory offices in vaccination centres. As an example, a large number of municipalities have set up an improvised eID application offices near the vaccination centres, inviting people to apply for smsPASS mobile digital identity on the way. Coordinated actions of the Ministry of Health and the Ministry of Public Administration have succeeded to promote adoption of both electronic identification and eHealth services, thus contributing to digital literacy of the population [46]. On the other hand, the NIJZ has taken measures to ensure access to the DCC to those without digital skills [47]. Paper copies of the vaccination DCC were sent to all persons vaccinated till July 2021. Free hard copies were available at vaccination centres and testing sites. In addition, a network of public and private pharmacies has been offering issuance of the DCC hard copies, reaching the widest population.

Despite the severe shortage of human resources at the NIJZ, Slovenia launched the DCC on 23 June. This would not be possible without a sound and comprehensive eHealth infrastructure and eHealth expertise acquired since the introduction of the national eHealth services in 2015. The DCC activities were carried out by the most competent members of the eHealth team working on top of their regular workload according to each person’s particular expertise. Nevertheless, the NIJZ has embarked on the eHealth (and the DCC) planning, development and maintenance tasks with full responsibility and thorough consideration of not only source data integrity but also cybersecurity and privacy risks pertaining to such extensive processing of healthcare data [48,49]. Data protection impact assessment was drawn up as part of the project design documentation, positioning the DCC application in the wider information security network [50]. In accordance with the NIJZ’s sectoral information protection policies, all key organizational, technical, and logical measures are in place for preventing the unauthorized processing of personal data in line with the requirements referred to in the Personal Data Protection Act [51] and the General Data Protection Regulation (GDPR) [52].

The Slovenian DCC application allows easy modifications of business logics with no need to upgrade the core elements while harvesting the full potential of the pre-existing eHealth services. Furthermore, the connection to the national CRPD platform ensures maximum inclusion of the healthcare providers and accuracy of the data. The central DCC service ensures the medical-grade quality, and the DCC is available to the whole population. Operational experience demonstrates that the DCC architecture in Slovenia provides exceptional performance, robustness, and flexibility. According to the statistics and data reported by the EU Member States [53], Slovenia has one of the highest numbers of the DCC issued per capita.

Statistical data presented in Table 1 indicates that the introduction of the DCC correlates with the increased number of visits to the zVEM Patient Portal and Mobile Application. The upsurge of registered users confirms that the DCC was a powerful promotor of the usage.

**Figure 3:**
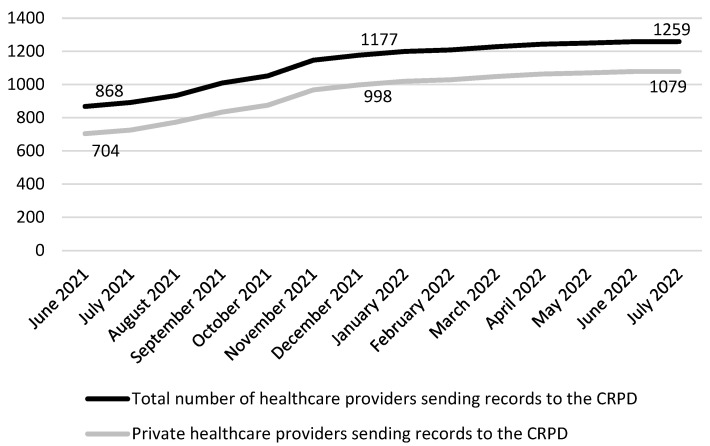
Growth in the number of healthcare providers sending records to the CRPD.

The number of healthcare providers sending documents to the CRPD has significantly increased in the observed period, mostly due to submission of the COVID-19 test results and other data related to COVID-19. There is a particularly significant upsurge in the number of private entities which were previously reluctant to participate in the national eHealth infrastructure (Figure 3).

Although the project of introducing the DCC at the EU level can be generally considered successful, it is necessary to point out certain issues that caused many complications and delays during the introduction and subsequent use of the DCC. Sudden and disruptive changes and decisions of the European Commission and the individual EU Member States, which concerned both the harmonization and the business rules for issuing and verification of the DCCs, seriously affected the development, maintenance, and usability of the DCC application.

The main problems arose mainly due to unpredictable and frequent changes in the issuing criteria, which triggered alterations of the key DCC parameters owing to emerging scientific findings about the disease, differences in medical doctrines, test market regulations, and diverse public health policies between the Member States. While the validity of the vaccination certificate was initially indefinite, it was later limited to 270 days and booster shots were required. The recovery certificate that was initially issued only upon the positive PCR test could later be issued on the basis of the RAT as well. In addition, the dynamics of the RAT market resulted in recurrent changes of the list of mutually recognized RATs, which were not efficiently communicated. Not only were new tests added frequently, some tests were also removed. The removal of the previously recognized RATs was particularly problematic not only due to confusion about the validity of the respected recovery certificates, but also due to financial and logistical troubles at test facilities. There were also discrepancies regarding the sequence of the vaccination dose and its recording, particularly in combinations of vaccinations and recovery events. Serious technical and legal issues appeared with the recognition of vaccinations and tests from the other Member States and third countries. Albeit understandable in the given emergency situation, such incoherent tactics and improvised solutions should be avoided by all means in future cross-border projects.

### 3.5. The National Legislation, the GDPR, and the European Health Data Space

The processing of personal data within all eHealth solutions including the DCC application is defined in the legislation, chiefly the Healthcare Databases Act [54], which delineates in detail the purpose and scope of data processing, lawful controllers and eligible users, sources of data, and linking between different databases. In accordance with the legal provisions, all key organizational, logical, and technical measures are in place for preventing the unauthorized processing of personal data in line with the requirements referred to in Articles 24 and 25 of the Personal Data Protection Act and Article 32 of the GDPR. Measures are in place to restrict access to data on the basis of user rights; all accesses and other forms of data processing are recorded with the help of audit trails; network traffic is adequately filtered, and data is protected by means of a centrally managed anti-virus protection system. The security measures are defined in detail in the “Overarching and sectoral information protection policies” published by the NIJZ.

Personal data within the eHealth information system databases is processed pursuant to the Healthcare Databases Act, which specifies the purpose and the scope of processing, the types of data processed and the data retention period. Individuals may exercise the rights deriving from the GDPR in relation to personal data processing via the publicly accessible contacts. The NIJZ has appointed a data protection officer (DPO) and deputy DPO who are tasked with resolving any claims relating to personal data protection and the rights derived from the GDPR. The NIJZ takes measures to inform individuals (either at the point of data capture, if possible, or via the website) on how and for what purposes data is collected; it also facilitates the exercise of rights relating to data protection under the GDPR. The NIJZ ensures that the data transfer process runs smoothly and facilitates that any special corrections to data are made on time, which also includes the prompt resolution of requests for changes to data under the GDPR. The NIJZ carries out regular audits of personal data processing procedures in order to improve data protection. These efforts include a data protection impact assessment (DPIA) for the eHealth information system. The purpose of carrying out the DPIA is to ensure the compliance of personal data processing with the requirements of the GDPR, to define and identify risks to the protection and security of personal data, and to determine measures to reduce these risks to an acceptable level, thereby adhering to the basic principles of the GDPR regarding personal data processing. As eHealth is the largest (central) health information system in Slovenia, and because it involves the extensive and systemic processing of personal health-related data, the DPIA is required not only because of the formal requirements set out in Article 35 of the GDPR, but also because of the actual security risks relating to unauthorized personal data processing.

The fact that GDPR is directly applicable in all Member States implies strong and harmonized data protection framework across the EU and strengthens mutual trust among the Member States. Joint controllership as stipulated in Article 26 of the GDPR has been applied to include all Member States participating in the DCC and is applicable in similar pan-European interoperability establishments. Similarly, the implementation of the EU Directive concerning measures for a high common level of security of network and information systems across the EU [55] has provided a legal ground for a sound and interconnected cybersecurity landscape. However, the Member States may still impose further constraints in national regulations on data protection and data processing in healthcare, which may impede harmonization and interoperability. Lack of alignment and interoperability in the EHR exchange hinders not only cross-border healthcare provision, but also response to cross-border public health threats, secondary use of health data and digital transformation of healthcare all over the EU. Recognizing these issues, the European Commission has recently published a proposal for the regulation on the European Health Data Space (EHDS Regulation) aiming to address legal, semantic, technical and political aspects of the EHR exchange and health data interoperability [56]. The scope of the EHDS Regulation is very comprehensive and demanding not only in terms of long-term financial and political obligations of the Member States and their digital health authorities, but also for the establishment of interoperable EU-wide cross-border digital infrastructure. Such infrastructures will need to be established for both primary EHR exchange for the purpose of medical care of individuals, and sharing health data for secondary use. Moreover, the EHDS Regulation provides obligations for commercial providers of the related digital systems to follow common standards through mandatory publications of technical specifications, conformance assessments and certifications. Accordingly, the EHDS Regulation aims to enforce interoperability of digital health applications and sharing of health data as a direct legal obligation in all Member States. The proposal is still in the negotiation phase and may be changed considerably before actually entering into force. However, it is already clear that the implementation will be very challenging. Besides substantial funding, profound political and organizational changes will be needed in healthcare systems. In addition, healthcare provides will need not only to invest in standardization of digital systems, but also to adjust the daily healthcare processes in order to provide the EHR records in a required manner. Although the European Commission and the Member States have high hopes for the long-awaited standardization in digital health, the results of the EHDS Regulation are far from granted, and a lot of hard work will need to be done by all stakeholders to achieve the declared objectives.

## 4. Discussion

Experiences from the COVID-19 pandemic have proven that the pre-existing eHealth infrastructure is indispensable in coping with public health emergencies [57,58]. Statistical data undoubtedly indicate that healthcare professionals and patients are becoming increasingly aware of the value of digital health, particularly in stressful situations that profoundly affect the operation of the healthcare system [59,60]. A particularly high interest was encountered in relation to microbiology laboratory test reports, COVID-19 test results and vaccination records, recovery certificate, and especially the DCCs as confirmed by the national DCC statistics as well as studies in other countries [2,42,44,61,62,63].

From the technological, process, and even political point of view, the EU DCC project was a great success, and on the deadline of 1 July 2021 the DCC was implemented not only by all Member States but also by many third countries (67 countries altogether in June 2022). Besides the regulation and substantial financial funding, the timely publication of technical specifications and development of central digital platform and building blocks (EU Gateway) must be credited. In addition, the project was facilitated by intense coordination of international expert communities within the eHealth Network [64].

However, the national implementations were not fully harmonized, leading to inconsistencies and consequent unpredictability in certain areas [65,66]. This is rather expectable and understandable, since the whole project was based on searching for the lowest common denominator of the EU institutions, the EU Member States, and healthcare authorities (national and international). Healthcare, including the area of communicable diseases, is the responsibility of the EU Member states, and epidemiological measures have been primarily focused on the national epidemiological situations and specifics [67,68]. Despite the lengthy harmonization process, public health policies across the EU are not fully unanimous and the Member States have kept some degree of autonomy in setting the national rules for cross-border entry. This further complicated the adoption of the matching DCC applications at the level of EU and brought substantial confusion and annoyance amid the travelling citizens who were struggling to follow the frequently changing rules.

The whole concept and implementation of the DCC are rather complex due to the numerous and relatively distributed data sources, on one hand [69,70], and the complex dependencies and business rules on the other. Irrespective of the enormous public and political pressure and the disturbing conditions in which the DCC project was implemented [71,72], it should be emphasized that the rapidly changing business rules on issuance and verification caused the majority of problems and setbacks in the development, maintenance, and everyday use of the DCC application. Poor quality and integrity of the collected data due to irregular or deficient reporting by healthcare providers, numerous amendments of the national regulations, frequent changes of the list of mutually recognized RAT test devices, lack of reliable data on citizens vaccinated and/or tested abroad, and predicaments in interpreting and articulating business rules in the diverse combinations of vaccination and test events are roughly the main problems that we stumbled upon in Slovenia. Poor coordination of policymakers and other stakeholders, the vaccine scepticism and citizen distrust, erratic and inefficient communication, and confusing information that has overflowed the media realm have only aggravated the aforementioned issues [73,74,75].

The case of establishing the DCC system showed that Slovenia has a well-developed, reliable and scalable eHealth infrastructure. The efficiency and adaptability of the eHealth infrastructure in Slovenia was largely achieved due to the efforts and investments over the past years, on the basis of which the central building blocks of eHealth were established. These central building blocks, including the CRPD, the Electronic Immunization Registry, the zVEM Patient Portal and the zVEMplus web portal, represent the key national platforms that enabled the collection of relevant data on vaccination and testing and without which it would not have been possible to establish the DCC system or to issue DCCs in a controlled and unified manner. The establishment of the entire DCC system was based on the integration of these central building blocks via the web services. As a result, the development of the DCC was less demanding because the newly introduced DCC issuing application only needed to incorporate the business rules into the operational logic. A centralized approach to building a national eHealth architecture has repeatedly proven to be very useful in Slovenia, as it enables good control, rapid adjustments and integration of new components, as well as comprehensive access to relevant data collected at the national level. In addition, it simplifies integration with the DCC infrastructure as the NIJZ is the sole competent issuer and signatory.

Slovenia is relatively small in terms of geographic area and population (approx. 2 million inhabitants), which facilitates such an approach. However, we believe that a nationally led and centralized approach to building eHealth infrastructure is much more challenging in larger countries, which are faced with administrative divisions, a larger and more complex structure of the healthcare system, divided powers and responsibilities between local and regional authorities and the state. Nevertheless, it must be emphasized that both the centralized and regional approaches have certain advantages and disadvantages, which largely depend on the policy, management, organizational, financial, infrastructural, and other aspects.

To sum it up in general terms, the use of the DCC was widespread and the usage of the zVEM Patient Portal and Mobile Application has increased immensely. Citizens and healthcare professionals obviously recognized the value of public digital services, as the growth in adoption and usage also revealed the rise of confidence in national eHealth services [76,77]. Regardless of positive experiences in some areas, the efficacy of the DCC and related approaches to limit the spread of COVID-19 infections will need to be meticulously documented, analysed, and objectively evaluated [78,79]. Besides the epidemiological impacts, ethical concerns, professional and scientific dilemmas, and global technological feasibility, wider implications of the DCC and similar socio-technical measures are definitely the issues which should be addressed as a matter of priority in future research in this field [80,81,82].

## 5. Conclusions

Reaching the firm political agreement between the Member States and development and adoption of common technical specifications at the EU level with clearly defined rules and protocols for the integration of national digital infrastructures are the most important gains of the DCC project. The lessons learned, compelling experience from the establishment of the DCC, and evidence collected from the other Member States could provide a commonly applicable model for the forthcoming conceptualization, design, development, and rollout of cross-border eHealth services in the EU. The cross-border cooperation of health authorities is essential to pandemic management and bringing the pandemic under control, and similar applies for the governance of the supporting digital services. Moreover, the DCC outcomes contributed greatly to the promotion of digital health in the Member States and strengthened the political commitment for the future investments in digital health at the European Commission. This is particularly important for the planned implementation of a large-scale strategic project such as the EHDS Regulation, which will require a lot of effort and resources at the EU level.

Nonetheless, the experience with the establishment of the DCC has revealed that all similar digital projects at the EU level necessitate a technologically advanced national digital infrastructures, including scalable and resilient digital health services and databases, an adequate number of proficient experts, clear data processing and cybersecurity rules, and, evidently, sufficient funding and adequate legal framework. The aforementioned prerequisites are indispensable not only for the successful implementation of digital health initiatives at the EU level, but also for widespread adoption of the existing and new national digital health services that are readily and trustfully accepted by citizens and healthcare providers. Without this in mind, such projects can turn into expensive and painful setbacks that fail to contribute to the achievement of common public health goals, and, what is worse, failed projects could have an adverse effect on all future initiatives for the development of national and cross-border eHealth solutions in the EU.

## Figures and Tables

**Table 1:** Dynamics of the DCC issuance, zVEM Patient Portal visits, and the zVEM registered users.

	DCCs Issued Monthly	zVEM Patient Portal Visits Monthly	zVEM Registered Users (Total End of Month)
May 2021		2,592,507	133,405
June 2021	1,841,263	3,806,710	249,958
July 2021	1,709,241	1,152,201	313,643
August 2021	1,019,596	2,264,642	336,770
September 2021	1,760,842	3,191,890	363,864
October 2021	1,247,155	2,101,672	375,928
November 2021	2,307,858	3,000,222	393,558
December 2021	2,926,062	2,460,899	406,892
January 2022	3,188,018	3,737,162	420,870
February 2022	2,167,248	2,943,059	431,383
March 2022	432,426	1,353,052	435,603
April 2022	322,236	1,065,575	439,020

## Data Availability

Data available on request due to restrictions eg privacy or ethical. The data presented in this study are available on request from the corresponding author. The data are not publicly available due to privacy issues.
